# Erythrocyte Shape Abnormalities, Membrane Oxidative Damage, and **β**-Actin Alterations: An Unrecognized Triad in Classical Autism

**DOI:** 10.1155/2013/432616

**Published:** 2013-12-21

**Authors:** Lucia Ciccoli, Claudio De Felice, Eugenio Paccagnini, Silvia Leoncini, Alessandra Pecorelli, Cinzia Signorini, Giuseppe Belmonte, Roberto Guerranti, Alessio Cortelazzo, Mariangela Gentile, Gloria Zollo, Thierry Durand, Giuseppe Valacchi, Marcello Rossi, Joussef Hayek

**Affiliations:** ^1^Department of Molecular and Developmental Medicine, University of Siena, Via A. Moro 2, 53100 Siena, Italy; ^2^Neonatal Intensive Care Unit, University Hospital, Azienda Ospedaliera Universitaria Senese (AOUS), Viale M. Bracci 16, 53100 Siena, Italy; ^3^Department of Life Sciences, University of Siena, Via A. Moro 2, 53100 Siena, Italy; ^4^Child Neuropsychiatry Unit, University Hospital, AOUS, Viale M. Bracci 16, 53100 Siena, Italy; ^5^Medicine, Surgery and Neurosciences Department, University of Siena, Viale M. Bracci 16, 53100 Siena, Italy; ^6^Clinical Pathology Laboratory Unit, University Hospital, AOUS, Viale M. Bracci 16, 53100 Siena, Italy; ^7^Department of Medical Biotechnologies, University of Siena, Via A. Moro 2, 53100 Siena, Italy; ^8^Institut des Biomolécules Max Mousseron (IBMM), UMR 5247, CNRS/UM1/UM2, BP 14491 34093, Montpellier, Cedex 5, France; ^9^Life Science and Biotechnologies, University of Ferrara, Via Borsari 46, 44100 Ferrara, Italy; ^10^Department of Food and Nutrition, Kyung Hee University, 1 Hoegi-dong, Dongdaemun-gu, Seoul 130-701, Republic of Korea; ^11^Respiratory Pathophysiology and Rehabilitation Unit, University Hospital, AOUS, Viale M. Bracci 16, 53100 Siena, Italy

## Abstract

Autism spectrum disorders (ASDs) are a complex group of neurodevelopment disorders steadily rising in frequency and treatment refractory, where the search for biological markers is of paramount importance. Although red blood cells (RBCs) membrane lipidomics and rheological variables have been reported to be altered, with some suggestions indicating an increased lipid peroxidation in the erythrocyte membrane, to date no information exists on how the oxidative membrane damage may affect cytoskeletal membrane proteins and, ultimately, RBCs shape in autism. Here, we investigated RBC morphology by scanning electron microscopy in patients with classical autism, that is, the predominant ASDs phenotype (age range: 6–26 years), nonautistic neurodevelopmental disorders (i.e., “positive controls”), and healthy controls (i.e., “negative controls”). A high percentage of altered RBCs shapes, predominantly elliptocytes, was observed in autistic patients, but not in both control groups. The RBCs altered morphology in autistic subjects was related to increased erythrocyte membrane F_2_-isoprostanes and 4-hydroxynonenal protein adducts. In addition, an oxidative damage of the erythrocyte membrane **β**-actin protein was evidenced. Therefore, the combination of erythrocyte shape abnormalities, erythrocyte membrane oxidative damage, and **β**-actin alterations constitutes a previously unrecognized triad in classical autism and provides new biological markers in the diagnostic workup of ASDs.

## 1. Introduction

Autism spectrum disorders (ASDs) are considered to be the result of a complex interaction between a genetic background and environmental factors [1, 2]. Autism is a heterogeneous, behaviorally defined neurodevelopmental disorder affecting four times more males than females [3], with a clinical onset usually within the 2nd year of life and mainly consisting of social impairment; communication difficulties; and restricted, repetitive, and stereotyped patterns of behavior. Classical autism is the overwhelmingly predominant ASDs phenotype, which also include other four disorders, that is, Asperger syndrome and pervasive developmental disorder not otherwise specified (PDD-NOS), and childhood disintegrative disorder. Rett syndrome (RTT) is a genetically determined neurodevelopmental disorder with autistic features, and has been recently separated by the ASDs as a distinct nosological entity (Diagnostic and Statistical Manual of mental disorders V-DSMV).

The search for specific and reliable biomarkers is of paramount importance in autism, given its dramatically rising prevalence in the general population over the last two decades [4] from 1 in 5000 in the mid-1970s to 1 in 88 in 2008 [4, 5].

A redox imbalance has been repeatedly reported in autism [6–8] although its role in the pathogenesis is still under question. In particular, it is unclear whether oxidative stress (OS) is a cause or consequence of autism [9]. Recent studies indicate that autism is associated with deficits in glutathione antioxidant defense in selective regions of the brain, thus potentially contributing to OS, immune dysfunction, and apoptosis, particularly in the cerebellum and temporal lobe [10].

More recently, OS and erythrocyte membrane alterations have been described in autistic children, including elevated erythrocyte thiobarbituric acid reactive substances, urinary isoprostane, and hexanoyl-lysine levels, associated with a significant reduction of Na^+^/K^+^-ATPase activity, a reduction of the erythrocyte membrane fluidity and an alteration in the erythrocyte fatty acid membrane profile linked to an increase in monounsaturated fatty acids, a decrease in eicosapentaenoic acid and docosahexaenoic acid, and a consequently increased *ω*-6/*ω*-3 ratio [11].

The peculiar triad of OS imbalance, mild chronic hypoxia, and an abnormally high frequency of leptocytes in the peripheral blood has been reported by our group in girls RTT [12], a relatively rare neurodevelopmental disorder almost affecting females and mainly due to *de novo* mutations of the X-linked methyl-CpG-binding protein 2 (*MeCP2*) gene [13]. These data indicate that redox imbalance and oxygen exchange could be key players in the pathogenesis of this particular human model of autism.

The shape is critical to red blood cells (RBCs) function and blood rheological properties, and emerging evidence indicates that OS is a key factors in modulating erythrocyte shape [14–16]. Although RBCs membrane lipidomics [11] and rheological variables [17] have been reported to be altered, and some suggestions of an increased lipid peroxidation in the erythrocyte membrane exist [11], to date no information is available on how the RBCs oxidative membrane damage may affect cytoskeletal membrane proteins and, ultimately, erythrocyte shape in autism.

In the present study, we investigated RBC morphology, *in situ* membrane oxidative damage, and cytoskeletal proteins in patients with classic autistic disorder.

## 2. Methods

### 2.1. Subjects Population

A total of *n* = 15 patients (male: 9; female: 6), with classic autistic disorder (mean age at examination: 15.9 ± 5.9 years, range 6–26), as well as *n* = 15 healthy controls of comparable age and a typical neurodevelopment (mean age: 16.3 ± 6.2 years, range 5–30; male: 8; female: 7) participated in the study. Furthermore, a third group of 15 patients (male: 7; female: 8) with nonautistic neurodevelopmental disorders (NA-NDDs) (mean age at examination: 15.8 ± 6.0 years, range 5–28) was enrolled as a “positive control” population, including idiopathic mental retardation (*n* = 6), cerebral palsy (*n* = 3), Attention-Deficit/Hyperactivity Disorder (ADHD) (*n* = 4), and language disorders (*n* = 2). Childhood Autism Rating Scale scores (CARS) [18] for the examined autistic patients and the NA-NDDs groups were estimated. The populations of patients and healthy subjects were recruited by the medical staff of the Child Neuropsychiatry Unit of the Azienda Ospedaliera Senese (Siena, Italy). All the enrolled patients and healthy subjects were genetically unrelated. The autistic patients were diagnosed by DSMV and evaluated using Autism Diagnostic Observation Schedule (ADOS), and Autism Behaviour Checklist (ABC). Patients with RTT, X-fragile syndrome, or tuberous sclerosis, as well as subjects with clinical evident sideropenic anemia, perinatal adverse events, and/or brain abnormalities on magnetic resonance imaging, were excluded for the present study. All subjects were on a typical Mediterranean diet.

The study was conducted after the approval by the Institutional Review Board and all written informed consents were obtained from either the parents or the legal tutors of the enrolled patients.

### 2.2. Routine Hematological Analyses

For these particular laboratory determinations, samples collected in tubes with K_2_EDTA were analyzed by an automated hematology system Sysmex XE-2100 (Sysmex corporation, Japan) in the automated aspiration (i.e., closed) sampling mode, using 200 *μ*L sample volume. The instruments are in routine use for count blood cells and automated differential counts analyses and underwent periodic quality assessment in internal and external control programs. A 5-part differential count was performed by lysing erythrocytes and analyzing the light scatter/fluorescence [19]. Blood smears were stained with standard May-Grünwald Giemsa within 6 hours after blood sampling (SP1000 instrument) and visualized by an automated image recognition system CellaVision DM96, an automated microscope with software showing digitalized images of the blood smears.

### 2.3. Blood Sampling for Erythrocyte Oxidative and Shape Analysis Studies

For these aims, an aliquot of blood was collected in heparinized tubes, and manipulations were carried out within 2 hrs after sample collection. Blood samples were centrifuged at 2400 ×g for 15 min at 4°C, whereas the platelet poor plasma and the buffy coat were removed by aspiration. RBCs were washed twice with physiological solution (150 mM NaCl). An aliquot of packed erythrocytes was resuspended in Ringer solution (125 mM NaCl, 5 mM KCl, 1 mM MgSO_4_, 32 mM N-2-hydroxyethylpiperazine-N-2-ethanesulfonic acid (HEPES), 5 mM glucose, 1 mM CaCl_2_), pH 7.4 as a 50% (vol/vol) suspension for the determination of intraerythrocyte nonprotein-bound-iron (NPBI). The remaining volume of packed RBCs was used for erythrocyte membranes preparations (i.e., hemoglobin-free ghosts) for 4-hydroxynonenal protein adducts (4-HNE PAs) determinations.

An aliquot of each blood sample (1 mL) was centrifuged at 800 ×g for 10 minutes at 4°C for scanning electron microscopy (SEM) analysis of erythrocytes.

### 2.4. Scanning Electron Microscopy (SEM)

As previously described [12], erythrocytes were plated on poly-l-lysine coated slides and fixed in Karnowsky (2.5% glutaraldehyde—4% paraformaldehyde in 0.1 M sodium-cacodylate buffer, pH 7.2) for 2 h at 4°C, rinsed twice for 10 min with 0.1 M sodium cacodylate buffer and postfixed in 1% osmium tetroxide in 0.1 M sodium-cacodylate buffer for 1 h at 4°C. Specimens were then dehydrated through a graded ethanol series, dried in a CO_2_ critical point dryer (CPD010, Balzers Union, Liechtenstein), mounted on specimen stub, sputter coated with gold (Sputter Coater S150B, Edwards, England), and examined in a XL 20 SEM (Philips, Eindhoven, Netherlands). Altered RBCs shapes were evaluated by counting ≥800 cells (50 erythrocytes for each different SEM field at a magnification of ×3000) from all groups of subjects. All countings were carried out in triplicate and averages were taken for data analysis.

### 2.5. Intraerythrocyte NPBI

Intraerythrocyte NPBI (nmol/mL) was determined as a desferrioxamine (DFO)-iron complex (ferrioxamine), as previously reported [20]. Briefly, 25 *μ*M DFO was added to the samples (1 mL erythrocyte suspension), the erythrocytes were then lysed by adding water (1 vol) and freezing (−70°C) and thawing. The hemolysate was ultrafiltered at 3373 ×g for 30 min in centrifugal filters with a 30 kDa molecular weight cutoff (VIVASPIN 4, Sartorius Stedim Biotech GmbH, Goettingen Germany) and the ultrafiltrate was stored at −20°C until analysis. The DFO excess was removed by silica (Silicagel; 25–40 *μ*m) column chromatography. The DFO-iron complex was determined by HPLC and the detection wavelength was 229 nm. The calibration curve correlation for intraerythrocyte NPBI was adequate (*r* ^2^ = 0.994009), the minimum detection limit was 0.1 nmol/mL, and mean intra- and inter-observer coefficients of variation were ≤2.5% and ≤5%, respectively.

### 2.6. Erythrocyte Membrane Preparation

An aliquot (600 *μ*L) of packed RBCs was lysed in Dodge buffer, and erythrocyte membranes were prepared, according to Dodge et al. [21], by repeated washing until the “ghosts” were pearly white. Samples were kept frozen at −70°C until used for sodium dodecyl sulfate-polyacrylamide gel electrophoresis (SDS-PAGE), immunoprecipitation (IP), and esterified F_2_-isoprostanes (F_2_-IsoPs).

### 2.7. Immunoprecipitation of Erythrocyte *β*-Actin

Erythrocyte membrane (ghosts) proteins (200 *μ*g) were incubated with 5 *μ*g *β*-actin antibody (Millipore Corporation, Billerica, MA, USA) overnight at 4°C on a rotator. Then, immune complex was incubated with 50 *μ*L of Protein A-Sepharose (Sigma-Aldrich, Milan, Italy) and rotated at 4°C for 2 h. Samples were centrifuged at 10,000 g for 5 min and washed three times with 1 mL ice-cold PBS. The pellet was mixed with 2X reducing sample buffer, boiled, and loaded on SDS-PAGE gels for silver staining or western blotting analysis.

### 2.8. Silver Staining of Erythrocyte Membrane Proteins and Immunoprecipitated *β*-Actin

Erythrocyte membrane (i.e., “ghosts”) proteins (20 *μ*g protein, determined using BioRad protein assay; BioRad, Hercules, CA) or immunoprecipitated *β*-actin were separated by the polyacrylamide gel electrophoresis (one-dimensional) method for discontinuous SDS-PAGE on 10% polyacrylamide gels in denaturing conditions, according to Laemmli [22]. At the end of the electrophoretic run, gels were stained with silver nitrate for protein visualization (Sigma-Aldrich, Milan, Italy). Gel image was acquired using image scanner and the bands were automatically detected and analyzed using TotalLab software (nonlinear dynamics, version 1.0). Band volume was expressed as a ratio of the total protein volume detected from the entire gel to minimize differences between band (band normalization) and to compare band measurements in different lanes.

### 2.9. Western Blot for 4-HNE Protein Adducts in Erythrocyte Membrane Proteins and Immunoprecipitated *β*-Actin

Western blot protocols were performed as previously described [12]. Erythrocyte membrane proteins (40 *μ*g protein, determined using BioRad protein assay; BioRad, Hercules, CA) or the immunoprecipitated *β*-actin were resolved on 10% SDS-PAGE gels and transferred onto a hybond ECL nitrocellulose membrane (GE Healthcare Europe GmbH, Milan, Italy). After blocking in 3% nonfat milk (BioRad, Hercules, CA, USA), the membranes were incubated overnight at 4°C with goat polyclonal anti-4-HNE adduct antibody (cod. AB5605; Millipore Corporation, Billerica, MA, USA). Following washes in TBS Tween and incubation with specific secondary antibody (mouse anti-goat horseradish peroxidase-conjugated, Santa Cruz Biotechnology, Inc., CA, USA) for 1 h at RT, the membranes were incubated with ECL reagents (BioRad, Hercules, CA, USA) for 1 min. Images were digitized (ChemiDoc XRS, BioRad, Hercules, CA) and band optical densities were quantified using a computerized imaging system (Quantity One Imaging system).

### 2.10. Erythrocyte Membrane Esterified F_2_-Isoprostanes (F_2_-IsoPs)

A 100 *μ*L aliquot of the erythrocyte membrane samples was resuspended with H_2_O (0.9 mL) and 1 N KOH (500 *μ*L) was added for the basic hydrolysis. After incubation at 45°C for 45 min, the sample was acidified to pH 3 with HCl 1 N (500 *μ*L), spiked with tetradeuterated Prostaglandin F_2*α*_ (PGF_2*α*_-d_4_) (500 pg in 50 *μ*L of ethanol; Cayman, Ann Arbor, MI, USA), as internal standard, and extracted with 10 mL of ethyl acetate. The upper organic layer, obtained after centrifugation at 1000 ×g for 5 min, was applied onto an aminopropyl (NH_2_) cartridge (Waters, Milford, MA, U.S.A.) preconditioned with 10 mL of hexane. After derivatization the determination of F_2_-IsoPs was accomplished by gas chromatography/negative ion chemical ionization tandem mass spectrometry (GC/NICI-MS/MS) analysis [12].

Esterified F_2_-IsoPs were normalized for membrane proteins and quantified by BioRad protein assay (BioRad, Hercules, CA) using 0.2% Triton X-100 to dissolve the membranes. The measured ions were the product ions at *m/z* 299 and *m/z* 303 derived from the [M-181]^−^ precursor ions (*m/z* 569 and *m/z* 573) produced from 15-F_2_t-IsoPs (the most represented F_2_-IsoP isomer) and PGF_2*α*_-d_4_, respectively [12].

### 2.11. Statistical Data Analysis

All variables were tested for normal distribution (D'Agostino-Pearson test) and data were presented as means ± SD for normally distributed variables. Differences between groups were evaluated using independent-sample *t*-test (continuous normally distributed data), Mann-Whitney rank sum test (continuous nonnormally distributed data), chi-square statistics (categorical variables with minimum number of cases per cell ≥5) or Fisher's exact test (categorical variables with minimum number of cases per cell <5), one-way analysis of variance (ANOVA), Student-Newman-Keuls post hoc test or Kruskal-Wallis test. Associations between variables were tested by univariate regression analysis (Pearson's coefficients or Spearman's rho, as appropriate). The effects of small population sizes on possible type I (*α*)/type II (*β*) errors in the data interpretation were examined using a sampling size algorithm. A two-sided *P* < 0.05 was considered to indicate statistical significance, and the Bonferroni-corrected significance levels were used for multiple *t*-tests. The MedCalc version 12.1.4 statistical software package (MedCalc Software, Mariakerke, Belgium) was used.

## 3. Results

### 3.1. IQ and CARS Estimates

Estimated IQ and CARS values for the autistic group were 40.6 ± 12.80 (range 20–60), and 51.9 ± 7.0 (range 41–60), respectively, versus 63.0 ± 8.54 (values range 55–72) and 26.0 ± 2.2 (values range: 24–29), respectively, (*t*-test statistics 5.638 and −13.671, respectively; *P* value *s* < 0.0001).

### 3.2. Red Blood Cell Counts

Blood cell counts in patients were not significantly different from those of the control groups, with the single exception of a nonsignificant trend for MCHC, slightly lower in the autistic group (*P ≥ 0.0669) (Table 1). In particular, no laboratory signs of anemia in any of the three groups were evidenced. Nevertheless, statistical differences regarding Hb and MCV were detectable for the NA-NDDs group (Table 1). These findings would suggest a “relative microcytic anemia” in this latter population.

**Figure 1:**
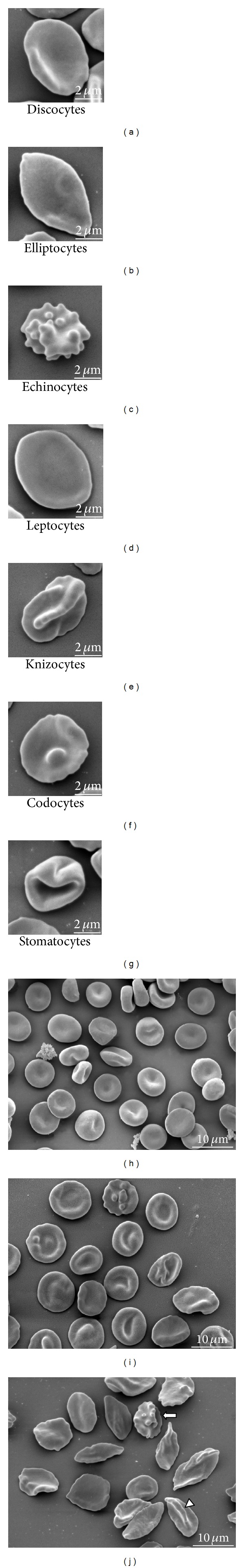
Abnormal erythrocyte shapes in classical autism at the scanning electron microscopy (SEM). (a): normal discocyte shape; (b) to (g): main shape-altered RBCs observed in autistic patients; (h): healthy controls; (i): a typical morphological pattern in nonautistic neurodevelopmental disorders (NA-NDDs); (j): typical picture in an autistic patient with predominant elliptocytosis. Symbols indicate intermediate-shaped RBCs: the arrow indicates a disco-echinocyte shape, while the arrowhead indicates the presence of a knizo-echinocyte shape in autistic patients, bars correspond to 2 *μ*m in (a) to (g) upper panels and to 10 *μ*m in the (h), (i), and (j) lower panels.

**Figure 2:**
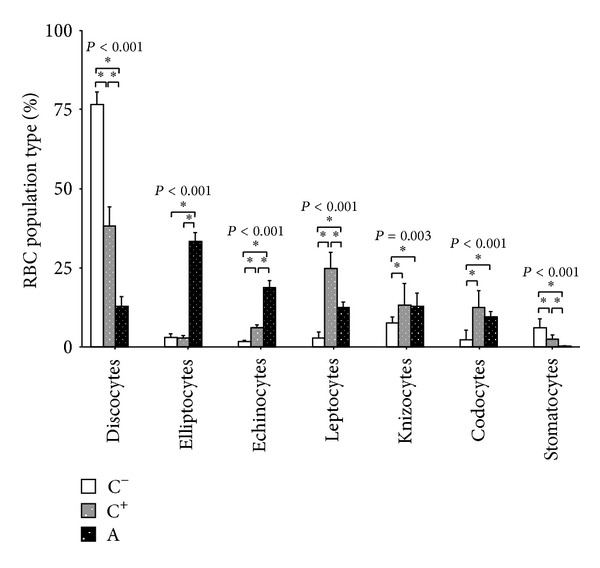
RBC morphology distribution in autistic patients, subjects with nonautistic neurodevelopmental disorders (NA-NDDs) and healthy controls. Statistically significant differences are denoted by single asterisk, *P* > 0.05. *P* values above each erythrocyte shape classification refer to one-way ANOVA.

At the SEM analysis, significantly higher percentages of altered RBCs shapes were present in the patients' groups, as compared to healthy control subjects (Figures 1 and 2). In particular, abnormally shaped erythrocytes in the blood samples from autistic subjects predominantly featured elliptocytes (33.2 ± 11.2% of all erythrocytes, range: 15.4–55.5). Besides elliptocytes, various abnormal RBC shape were observed, including echinocytes, leptocytes, knizocytes, codocytes, and stomatocytes.

The NA-NDDs group showed mixed abnormally shaped RBCs, without an overwhelmingly predominant erythrocyte shape phenotype, although a slight prevalence of leptocytes (~25%) was detectable.

Significant positive relationships were observed for the percentages of discocytes, leptocytes, or stomatocytes and RDW (*ρ* correlation coefficients range: 0.7245 to 0.8915). In addition, significant positive relationship were evidenced for the percentages of knizocytes and stomatocytes with Hb (*ρ* = 0.6907 and *ρ* = 0.5855, resp.), and the percentage of stomatocytes versus MCHC (*ρ* = 0.6070). Significant inverse relationships were found between percentages of codocytes and RDW (*ρ* = −0.8181), percentage of echinocytes and MCHC (*ρ* = −0.8379), and echinocytes versus Hb (*ρ* = −0.7776) (Table 2).

### 3.3. Erythrocyte Oxidative Status

**Figure 3:**
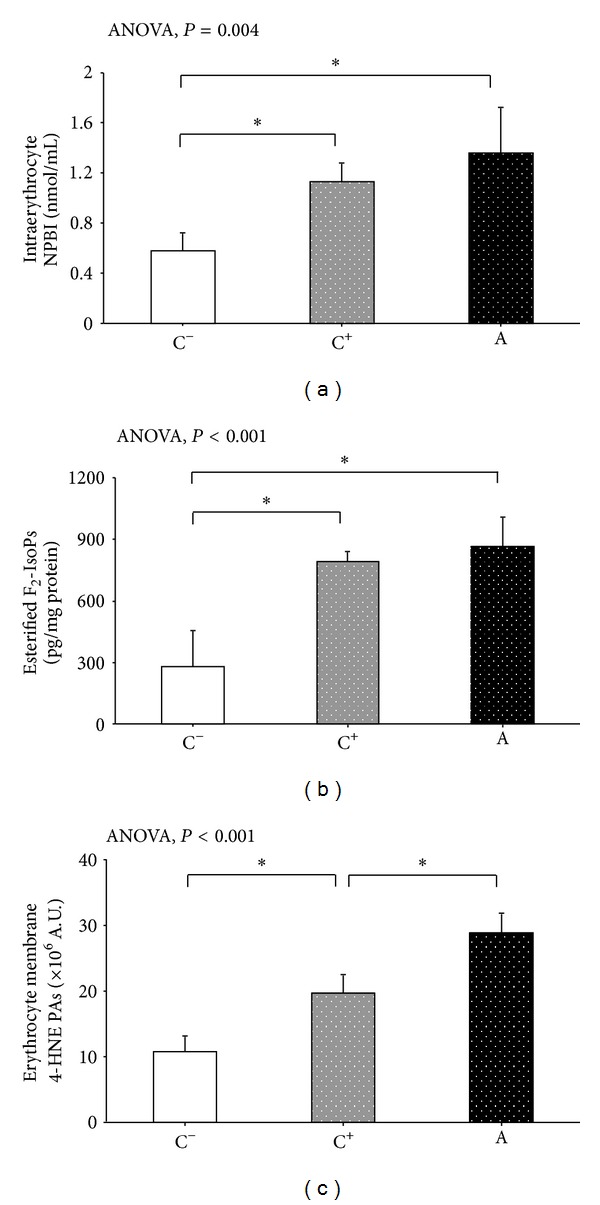
Intraerythrocyte NPBI, along with erythrocyte membrane esterified F_2_-IsoPs and 4-HNE protein adducts in autistic patients, subjects with nonautistic neurodevelopmental disorders (NA-NDDs), and healthy controls. NPBI was reported as nmol/mL erythrocyte suspension, esterified F_2_-IsoPs as pg/mg of erythrocyte membrane proteins, and 4-HNE protein adducts as arbitrary units. The data are expressed as means ± SD. Statistically significant differences are denoted by asterisks, *P* < 0.05. NPBI: non protein-bound-iron; F_2_-IsoPs: F_2_-isoprostanes; 4-HNE PAs: 4-HNE protein adducts.

Intraerythrocyte NPBI, together with erythrocyte membrane esterified F_2_-IsoPs and 4-HNE PAs, were significantly increased in autistic subjects, as well as patients with NA-NDDs (*F*-ratio range: 6.41 to 64.31; *P* value range: 0.001 to 0.004), as compared to healthy controls (Figure 3). A distinct 4-HNE PAs pattern was detectable (i.e., autism > NA-NDDs > healthy controls), thus indicating that classical autism and NA-NDDs are sharing erythrocyte oxidative membrane damage, although membrane proteins likely undergo different degrees of oxidation.

### 3.4. Erythrocyte Membrane Proteins

**Figure 4:**
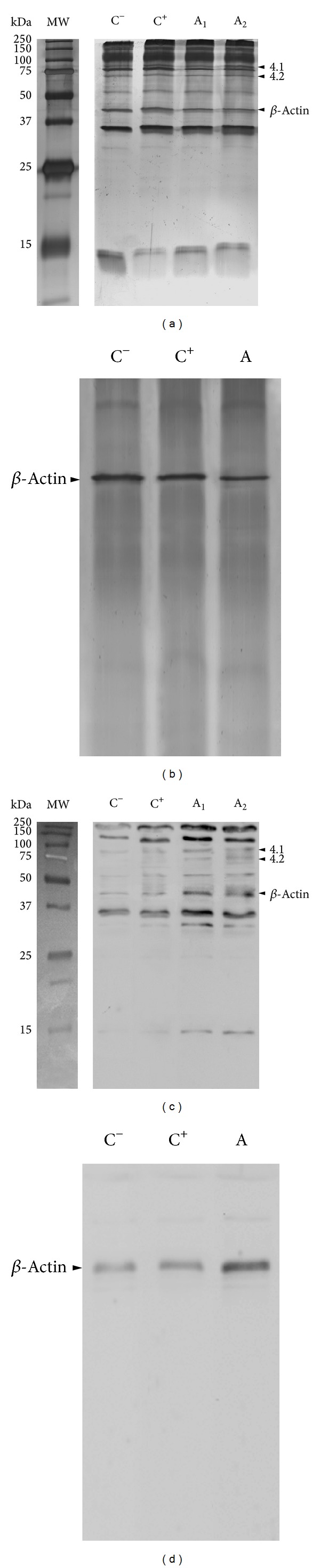
(a) Representative SDS-PAGE analyses of RBC ghosts (silver staining) in a healthy control subject (lane C^−^), a positive control subject (see text for definition; lane C^+^), and two autistic patients (lanes A_1_ and A_2_): visible reduction of intensity for *β*-actin in autistic patients (lanes A_1_ and A_2_) is evidenced; the electrophoretic position of bands 4.1 and 4.2 is indicated by arrowheads; (b) SDS-PAGE of immunoprecipitated *β*-actin (silver staining) from RBC ghosts of a healthy control subject (lane C^−^), a positive control individual (lane C^+^), and an autistic patient (lane A): visible reduction of intensity for *β*-actin in autistic patients (lane A); (c) immunochemical detection in the 4-HNE protein adducts in RBC ghosts: representative western blot from a healthy control subject (lane C^−^), a positive control individual (lane C^+^) and from an autistic patients (lanes A_1_ and A_2_). An increase in the 4-HNE PAs signal is evident in RBC ghosts from autistic patients (lanes A_1_ and A_2_), in particular as it concerns the *β*-actin band; the 4-HNE binding to bands 4.1 and 4.2 is indicated by arrowheads; and (d) immunochemical detection in the 4-HNE protein adducts in immunoprecipitated *β*-actin from RBC ghosts: representative western blot from a healthy control subject (lane C^−^), positive control individual (lane C^+^), and from an autistic patient (lane A): a visible increase in the 4-HNE PAs signal in *β*-actin from autistic patient (lane A) is evident. Molecular weight marks are indicated on the right side.

Typical SDS-PAGE analysis are shown in Figure 4(a). Alterations in the whole electrophoretic pattern of autistic patients were observed. In particular, a quantitative decrease in *β*-actin (−16 to −18.8%; *F*-ratio = 314.5; *P* < 0.001) was evidenced and further confirmed in the western blot analysis carried out after actin-IP by using a specific antibody (Figure 4(b)). In autistic patients the blotting study indicated detectable protein bands showing increased binding with 4-HNE, that is, the major aldehyde product of lipid peroxidation, as compared to both negative and positive control subjects (Figure 4(c)). *β*-actin was found to be a major target for 4-HNE binding, as indicated by densitometric analysis (+61.9 to +78.8%; *F*-ratio = 2622.8; *P* < 0.001) following *β*-actin IP (Figure 4(d)).

### 3.5. Correlations between Erythrocyte Shape, Membrane Oxidative Markers and *β*-Actin

The percentage of elliptocytes was positively correlated with IE-NPBI, erythrocyte membrane 4-HNE PAs, and esterified F_2_-IsoPs, as well as membrane 4-HNE *β*-actin adducts (4-HNE *β*-AAs) levels. The examined OS markers showed positive associations between them, while elliptocytes were inversely related to *β*-actin membrane content, and a remarkably high inverse relationship between amount of *β*-actin in the membrane and 4-HNE *β*-AAs was observed (*r* = −0.960; *P* < 0.001) (Table 3).

## 4. Discussion

Our findings show the coexistence of a peculiar combination of erythrocyte shape abnormalities, erythrocyte membrane oxidative damage, and *β*-actin alterations, which represents a previously unrecognized triad in classical autism.

### 4.1. RBCs Morphology

Several conditions, either congenital or acquired, are known to lead to abnormal erythrocyte shape [23]. The maintenance of the discoid shape is of paramount importance for the RBC main physiological role (i.e., transport of respiratory gases to and from tissues), while the deformability of the circulating cells has critical effects on the rheological properties of blood [24].

Our observations indicate that elliptocytes are the predominant abnormal erythrocyte shape occurring in the peripheral blood from patients with classical autism, and, to the best of our knowledge, this is the first time that an abnormal erythrocyte shape is reported in ASDs other than RTT. The presence of abnormal RBC shapes appears to be associated with an altered redox status in erythrocytes and a decrease and/or oxidization for some of the main cytoskeletal proteins known to be critical for the maintenance of the horizontal interactions in the erythrocyte membrane architecture. Although, abnormal RBC shape are reported in a series of associated conditions [23], this is likely the first time that an elliptocytosis is described outside of the known conditions of hereditary elliptocytosis and thalassemias [25]. Hereditary elliptocytosis is caused by weakened horizontal linkages in membrane skeleton due either to a defective spectrin dimer-dimer interaction or a defective spectrin-actin-protein 4.1R junctional complex. The severity of the disease is related to extent of decrease in membrane mechanical stability and resultant loss of membrane surface area. In contrast to the healthy population, in which elliptical RBCs shapes are up to 15% of erythrocytes, a diagnosis of elliptocytosis requires that at least 25% of erythrocytes in the specimen are abnormally elliptical in shape [25]. Therefore, it can be hypothesized that autism and hereditary elliptocytosis, an inherited blood disorder with an estimated incidence of 3 and 5 per 10,000 in the US population, may share common physiopathological mechanisms, although neither clinical signs of hemolytic anemia nor evidence of a shortened RBCs lifespan are known in autistic patients.

The NA-NDDs group, on the other hand, shows a heterogenous variety of abnormal RBC shape changes, with only ~40% of discocytes and a slightly dominance of leptocytes which appears to be just below 25% of all erythrocyte shapes in the blood samples. In this patient group, the SEM data were associated with a lower hemoglobin content and a higher degree of anisocytosis. Interestingly, we have previously described a marked (~55%) leptocytosis in *ω*-3 PUFAs unsupplemented girls with RTT [12], a neurodevelopmental disorder known to be the result of a single gene mutation [13]. In the near future, in our opinion, possible membrane cytoskeletal changes are to be carefully explored in this particular cause of cognitive impairment with autistic features.

Although iron deficiency in about a quarter of autistic children has been recently reported, resulting in iron deficiency anemia in about 15% of the cases and related to detective iron intake [26], none of our examined patients had clinically evident anemia. Interestingly, in a series of conditions, collectively termed “neuroacanthocytoses,” a distinctly abnormal erythrocyte shape (i.e., acanthocytosis) is reported to be associated with neurodegeneration and specific RBCs protein defects [27].

Genetic analyses over the past two decades have linked a full host of rare mutations to increased risk for ASDs, with a list of hundreds of autism risk loci in the human genome [28], whereas also common genetic variants are reported to affect the ASDs risk, but their individual effects appear to be modest [29]. Nevertheless, to the best of our knowledge, no genetic mutations involving the erythrocyte cytoskeleton have been previously described in autism.

### 4.2. Preliminary Assessment of the Relationship between RBC Biology and Autistic Behavior

Estimated IQ was significantly different between the two neurodevelopmental groups (i.e., autistic versus NA-NDDs), thus suggesting that the NA-NDDs group was actually composed by nonautistic patients with a demonstrated cognitive impairment, although of a less severe entity, as compared with that observed in the autistic population. Therefore, it is possible that stepwise changes in red blood cell phenotype between the autistic, positive, and negative control groups may be more closely linked to IQ than autism-specific differences. As a consequence, caution is needed before stating that an elliptic RBC shape is synonymous of autism, as we indicate that the triad “RBC shape + membrane OS damage + *β*-actin alteration” is associated with autism rather than elliptocytosis *per se.*

Furthermore, during our preliminary studies, we found that patients not currently included in the NA-NDDS nor in the autistic group showing mild “autistic like features” (i.e., a few patients with anorexia nervosa (nervous anorexia), currently classified as a psychiatric illness in the group of eating disorders and two patients with juvenile schizophrenia) showed an intermediate percentage of elliptocytes between the values here reported for the NA-NDDs and the control population (our unpublished data). Therefore, a proportionality between percentage of elliptocytes in the peripheral blood and autistic features in the behavior might be present, although, at the present time, we have no definite proof for it.

### 4.3. RBCs Cytoskeletal Proteins: *β*-Actin

Erythrocyte membrane integrity is critical for maintaining the erythrocyte characteristic shape and is based on both vertical and horizontal interactions among the cytoskeletal proteins, the integral membrane proteins, and the phospholipid bilayer. Vertical interactions are based either on spectrin, ankyrin, and band 3 protein or spectrin, 4.1 protein, and glycophorin, while horizontal interactions are mainly based on spectrin, 4.1 protein and actin [30–32]. In particular, *β*-actin is a globular protein composed of filaments, weakly binding to the tail end of *α* and *β* spectrins [33]. Our present findings evidence a deficiency in *β*-actin in the RBCs membranes from patients affected by classical autism, thus suggesting the coexistence of defective horizontal interaction forces in the cytoskeletal proteins of the erythrocyte membranes of these patients.

Besides the potential effects of *β*-actin deficiency on the membrane cytoskeletal structure of classical autistic patients, the detected *β*-actin alterations might be even more far-ranging given that emerging evidence indicated a role for *β*-actin in motor neuron function and axonal regeneration [34]. In particular, it has been suggested that distinct dynamics of Ca^2+^-*β*-actin could be a critical player in mediating the localized actin polymerization required for cellular constriction events mediating tissue bending, synaptic plasticity, and behavior [34, 35].

### 4.4. Potential Role of Exogenous or Endogenous Factor on the Erythrocyte Changes

Emerging evidence indicates that the role of environmental factors acting on genome by epigenetic regulation is relevant in autism and other neurodevelopmental diseases [36]. Moreover, a large number of toxic substances are known to cause erythrocyte damage in both experimental (i.e., phenylhydrazine and dapsone) [14, 37] and human settings (i.e., lead, penicillins, methyl-DOPA, and antiarrhythmics) [38], often leading to haemolytic anemias through different mechanisms. In our patients no clues exist for a specific exogenous molecule potentially causing an increased prevalence of elliptocytosis, and no evidence of haemolytic anemias was detectable. To date, no specific gene mutations involving the cytoskeletal components of the erythrocyte membrane have been demonstrate. Therefore, the demonstrated alteration of *β*-actin in our autistic patients appears to be the effect of a posttranslational modification, rather than the result of a specific gene mutation in the progenitor erythrocyte cells.

### 4.5. Oxidative Stress of the Erythrocyte Membrane

A link between oxidative stress and ASD has been previously reported by several authors [39–41]. Over the last few years, our team has demonstrated that OS is a key player in modulating the genotype-phenotype expression in RTT [20, 42–46].

Moreover, in previous reports, a correlation between OS and ASD has been widely explored by measuring different molecules, possibly coming from oxidative pathway as metabolic biomarkers of OS, in biological fluids [10, 39].

In autistic patients, the immunochemical detection of 4-HNE PAs indicate that oxidative events are ongoing in the RBCs of autistic patients and individuals with several neurodevelopmental disorders without autistic features. The reduction in membrane *β*-actin appears to be inversely related to the levels of 4-HNE *β*-AAs, thus indicating that the apparently reduced protein expression in the erythrocyte membranes from autistic patients is rather the consequence of a peculiar posttranscriptional modification linked to lipid peroxidation than the results of a reduced protein synthesis. This event, triggered by NPBI as a prooxidant factor [47], produces several compounds of degradation, 4-HNE among them. This highly reactive aldehyde can covalently bind proteins, phospholipids, and DNA; in particular, 4-HNE reacts readily with nucleophilic groups of amino acidic side chains, and its covalent attachment to proteins lead to alteration in their structure and biological activity [48]. Depending on its concentration and location, 4-HNE may be therefore considered as a “second toxic messenger,” which disseminates and augments initial free radical events.

Although a protective physiological role for OS—for instance oxidative shielding—has been recently underlined [49], there is little doubt that an oxidized protein is a damaged molecule with a likely reduced function. Thus, within the red cell membrane environment, oxidized proteins contribute to alter the phospholipid bilayer integrity and weaken the membrane mechanical properties, including a loss of the membrane fluidity as already reported in autism [6]. The report of significant alterations in the fatty acid profiles in individuals with ASDs in erythrocyte membrane [50] appears to be in line with our morphological and biochemical observations. Although an increased lipid peroxidation in the erythrocyte membrane from autistic patients has been suggested by using a thiobarbituric acid reactive substances (TBARs) assay kit [11], to date no further information on the *in situ* lipid and protein membrane damage is available. Our findings of increased erythrocyte membrane F_2_-IsoPs, as measured by a specific spectrometric method, and increased membrane 4-HNE-PAs reveal an increased arachidonic acid peroxidation, with a subsequent protein posttranslational modification in the erythrocyte membrane of patients with classical autism and NA-NDDs.

## 5. Conclusions

Our findings indicate the presence of an unrecognized triad combination of erythrocyte shape abnormalities, erythrocyte membrane oxidative damage, and *β*-actin alterations in classical autism and provides new biological markers in the diagnostic workup of ASDs. At least two unsolved questions are generated by our observations. Firstly, the specificity of our findings to autistic disorders is unknown to date and needs to be further explored. Secondly, the relationship between the abnormal erythrocyte shape and the neurological development in autistic children needs to be further investigated. The reported alteration in erythrocyte shape for classical autism could be theoretically translatable into a routine technology, such as fluorescence-activated cell sorting (FACS) either testing the volume or the morphological complexity of cells, in addition to the membrane fluidity.

In conclusion, our data shed new light on the concept of OS as a key factor in the pathogenesis of neurodevelopmental disorders. Interestingly, our data indicate that erythrocyte shape, either due to a defective RBC cytoskeletal scaffold or being the consequence of an oxidative cell damage, could be considered as a new potential physical biomarker for neurodevelopmental disorders.

## Figures and Tables

**Table 1:** Routine RBC variables in subjects with classical autism and nonautistic neurodevelopmental disorders (NA-NDDs) versus healthy controls.

RBC variables	Healthy controls	NA-NDDs	Classical autism	*P* value (ANOVA)
Hb (g/dL)	14.5 ± 0.9	13.6 ± 1.0*	14.2 ± 1.6	**0.001**
MCV (fL)	85.1 ± 6.6	83.3 ± 4.4*	86.1 ± 4.0	**0.004**
MCH (pg/cell)	28.8 ± 2.4	27.9 ± 1.6	28.6 ± 1.9	0.362
MCHC (g/dL)	33.9 ± 1.0	33.6 ± 0.9	33.2 ± 0.9	0.490
RDW (%)	13.4 ± 1.5	14.0 ± 2.1	13.1 ± 0.7	0.188

Data are expressed as means ± SD. *P value < 0.05 (post hoc analysis). Bold character indicates statistically significant differences.

Hb: hemoglobin; MCV: mean corpuscular volume; MCH: mean corpuscular hemoglobin; MCHC: mean corpuscular hemoglobin concentration; RDW: red cell distribution width.

**Table 2:** Correlation matrix for RBC shape as a function of RBC parameters in autistic patients.

RBC variables	Erythrocyte shape class						
	Disco-	Ellipto-	Echino-	Lepto-	Knizo-	Codo-	Stomato-
Hb	0.0159	0.1831	−0.7776*	0.1151	0.6907*	−0.0336	0.5855*
	(0.5452)	(0.9729)	(**0.0011**)	(0.4698)	(**0.0062**)	(0.9864)	(**0.0278**)
MCV	−0.2616	0.2063	−0.0556	−0.2841	0.2308	0.1960	0.4090
	(0.1479)	(0.2834)	(0.8471)	(0.2442)	(0.3414)	(0.4069)	(0.1580)
MCH	−0.0771	0.2980	−0.3959	−0.1329	0.2868	0.0847	0.4720
	(0.6423)	(0.3586)	(0.1732)	(0.4530)	(0.1144)	(0.8030)	(0.1099)
MCHC	0.2571	0.3688	−0.8379*	−0.0232	0.2720	−0.1233	**0.6070∗**
	(0.3502)	(0.1553)	(**0.0002**)	(0.8842)	(0.2860)	(0.3714)	(**0.0214**)
RDW	0.8915*	−0.0476	−0.4498	0.7245*	0.0357	−0.8181*	0.8278*
	(**0.0050**)	(0.8806)	(0.0672)	(**0.0078**)	(0.9516)	(**0.0038**)	(**0.0031**)

Data are Spearman's rho correlation coefficients with in brackets *P* values (*N* = 15). Bold characters with asterisks indicate statistically significant correlations.

Disco-: discocytes; Ellipto-: elliptocytes; Echino-: echinocytes; Lepto-: leptocytes; Knizo-: knizocytes; Codo-: codocytes; Stomato-: stomatocytes; Hb: hemoglobin; MCV: mean corpuscular volume; MCH: mean corpuscular hemoglobin; MCHC: mean corpuscular hemoglobin concentration; RDW: red cell distribution width.

**Table 3:** Correlation matrix for elliptic erythrocyte shape, oxidative stress markers, and *β*-actin erythrocyte membrane content.

	Elliptocytes	4-HNE PAs	IE-NPBI	F_2_-IsoPs	*β*-Actin content	4-HNE *β*-Actin adducts
Elliptocytes		0.779 [0.630–0.873] *P* < 0.0001	0.334 [0.045–0.572] *P* = 0.00246	0.588 [0.356–0.752] *P* < 0.0001	−0.860 [−0.921 to −0.758] *P* < 0.0001	0.887 [0.803–0.937] *P* < 0.0001
4-HNE PAs			0.397 [0.117–0.618] *P* = 0.0068	0.774 [0.622–0.869] *P* < 0.0001	−0.886 [−0.936 to −0.801] *P* < 0.0001	0.889 [0.806–0.938] *P* < 0.0001
IE-NPBI				0.415 [0.139–0.632] *P* = 0.0045	−0.414 [−0.631 to –0.138] *P* = 0.0046	0.340 [0.052–0.576] *P* = 0.0223
F_2_-IsoPs					−0.628 [−0.778 to −0.410] *P* < 0.0001	0.579 [0.344–0.746] *P* < 0.0001
*β*-Actin content						−0.960 [−0.978 to –0.929] *P* < 0.0001
4-HNE *β*-Actin Adducts						

Data are Spearman's rho correlation coefficients (in square brackets are the 95% confidence intervals for regression).

IE-NPBI: intraerythrocyte nonprotein-bound-iron; F_2_-IsoPs: F_2_-isoprostanes; 4-HNE Pas: 4-HNE protein adducts.
